# Relationship between cardiorespiratory fitness and arterial health in young-, and middle-age women: A mediation effect of body composition

**DOI:** 10.1016/j.jesf.2025.07.004

**Published:** 2025-07-16

**Authors:** Jitanan Laosiripisan, Napasakorn Chuensiri, Prin Ongkeaw, Thanonwat Sriputsayathanoth, Sawitree Poonpetpradab, Pornpicha Narmgate

**Affiliations:** a Department of Physical Therapy, Faculty of Allied Health Sciences, Thammasat University, Thailand; b Area of Exercise Physiology, Faculty of Sports Science, Chulalongkorn University, Thailand

## 1 Introduction

Cardiovascular diseases (CVD) remain a leading cause of dead worldwide.^1^ Arterial health plays a central role in the pathogenesis and progression of CVD. Two major types of arterial abnormalities that contribute to CVD risk include arterial stiffening^2^^,^^3^ and arterial narrowing. Both forms for vascular dysfunction compromise hemodynamic stability, increase cardiac workload, and promote atherosclerotic progression, ultimately elevating the risk of myocardial infarction, stroke, and other cardiovascular events.

Arterial stiffness refers to the loss of elasticity in the arterial wall. Arterial wall stiffness may be influenced by endothelial function through its regulation of smooth muscle tone (i.e., functional change) or change in the extracellular matrix structure (i.e., structural change).^4^^,^^5^ This stiffening impairs the artery's ability to buffer pulsatile blood flow, leading to increased systolic blood pressure, widened pulse pressure, and augmented cardiac afterload. A widely used non-invasive measure of arterial stiffness is pulse wave velocity (PWV)–specifically, brachial-ankle PWV (baPWV) or carotid-femoral PWV (cfPWV). PWV quantifies the speed at which pressure waves move through the arterial tree; higher velocity indicates greater stiffness. While PWV is clinically valuable due to its strong association with CVD risk, it can be influenced by transient factors such as heart rate and blood pressure, and baPWV in particular may be affected by muscular artery tone which in turn limits it specificity for central arterial stiffness.

In contrast, arterial narrowing refers to the reduction in arterial lumen diameter due to atherosclerotic plaque accumulation or vascular remodeling. This abnormality is often evaluated using carotid intima-media thickness (cIMT) and the ankle-brachial index (ABI). cIMT is measure via ultrasound as the combined thickness of the intima and media layers of the carotid artery and is used as a surrogate marker of subclinical atherosclerosis.^6^^,^^7^ cIMT primarily reflects structural changes in the arterial wall rather than functional impairment.

Among cardiovascular risks, evidence of excess adiposity and poor cardiorespiratory fitness (CRF) have been extensively acknowledged about negative effects on vascular function. Body composition significantly influences vascular function, with various measures of adiposity linked to impaired arterial health. Body mass index (BMI), although commonly used, does not differentiate between fat and lean mass or indicate fat distribution. Higher BMI has been associated with increased blood pressure,^8^^,^^9^ However, an increased arterial stiffness measured as carotid-femoral pulse wave velocity with an increased BMI has demonstrated a sex different association.^10^ Recent study in young population by Kalegna C.Z. et al. reported that carotid-femoral pulse wave velocity was associated with BMI in young men but not young women. However, the augmentation index, a surrogate of wave reflection pressure which affected by stiffness of the artery, was demonstrated a significant association with BMI in women but not in men.^10^ The most feasible link between obesity and obesity-related metabolic dyslipidemia might be the insulin resistance.^11^^,^^12^ Both insulin resistance and metabolic dyslipidemia are related to the pathological change of adipose tissue, the renin-angiotensin-aldosterone system, and sympathetic tone^13^, ^14^, ^15^ which in turn adversely affect arterial health.

Another body composition that more precisely reflects the fat distribution in the body is visceral adipose tissue (VAT). VAT is highly metabolically active and secretes a range of pro‐inflammatory cytokines and adipokines^16^, ^17^, ^18^, ^19^ such as tumor necrosis factor‐α (TNF‐α) and interleukin‐6 (IL‐6) which lead to chronic systemic inflammation, endothelial dysfunction, and reduced nitric oxide (NO) bioavailability. These changes lead to vascular dysfunction including arterial stiffness, higher systolic blood pressure which elevate risks for atherosclerosis and other cardiovascular diseases.

CRF, commonly measured via maximal oxygen consumption, is widely recognized as a powerful predictor of cardiovascular and metabolic health.^20^, ^21^, ^22^, ^23^, ^24^ It is widely acknowledged that the improvement in aerobic capacity could mitigate the arterial dysfunction.^25^, ^26^, ^27^, ^28^ Beneficial effects of CRF extend across a range of physiological systems, including improvements in endothelial function,^29^^,^^30^ arterial compliance,^31^^,^^32^ and inflammation regulation.^33^ One particularly important mechanism by which CRF exerts these benefits is through enhanced fat metabolism. Regular aerobic exercise increases mitochondrial density and oxidative enzyme activity in skeletal muscle, which improves the body's ability to mobilize and oxidize fatty acids—ultimately reducing fat accumulation, especially in metabolically active depots such as visceral adipose tissue. At the same time, reductions in visceral fat mass leads to a decline in inflammatory cytokine release, decreasing systemic inflammation. Beneficial effects of CRF are also demonstrated in the protective effects against vascular deterioration. Enhanced shear stress from regular exercise upregulates eNOS and promotes nitric oxide production, improving endothelial function. Exercise also strengthens endogenous antioxidant capacity, limiting NO degradation by reactive oxygen species.^34^, ^35^, ^36^ Collectively, these adaptations contribute to reduced arterial stiffness, as reflected in lower brachial-ankle pulse wave velocity and pulse pressure, highlighting the role of fitness in maintaining arterial health.

Given these interrelated benefits, understanding the mediating role of body composition, particularly parameters that could reflect fat accumulation (e.g., BMI, percentage of body fat, and visceral adipose tissue mass), may offer novel insights for tailoring aerobic exercise interventions. Such an approach could help shift from generic fitness recommendations toward precision-based exercise prescriptions that specifically target metabolic dysfunction and vascular risk.

Although individuals in midlife are not exempt from cardiovascular risk, recent trends indicate a concerning rise in coronary artery disease among younger and middle-age women,^37^ particularly a global populations continue to age. Recent epidemiological evidence suggests that women may be more vulnerable to the vascular consequences of excess body fat, women exhibited greater increases in blood pressure and arterial stiffness in response to weight gain compared to men.^38^ This greater susceptibility may reflect sex-specific differences in adipose tissue distribution and its metabolic activity.

Importantly, while estrogen confers some vascular protection, especially through its effects on endothelial function and nitric oxide production,^39^ this protection can be diminished or overridden by the presence of low cardiorespiratory fitness and obesity. Women with poor fitness and excess adiposity appear to experience exaggerated inflammatory responses, which accelerate vascular aging and elevate cardiovascular disease risk.^33^ Despite this, women remain underrepresented in research on cardiorespiratory fitness, body composition, and vascular health. Therefore, investigating these interrelations specifically in women is critical to developing targeted preventive strategies and improving cardiovascular outcomes in this high-risk population.

To bridge the research gaps mentioned above this study aimed to determine the mediating effects of body composition on the relationships between cardiorespiratory fitness and indices of arterial health (i.e., brachial systolic blood pressure; brSBP, brachial pulse pressure; brPP, baPWV, cIMT, and ABI) in premenopausal women. We hypothesized that body composition serves as a mediator in the relationship between CRF and arterial health.

## 2 Method

### 2.1 Participants

Female participants (n = 67) aged 18–45 years were recruited for the study. Inclusion criteria were: (1) a BMI between 18 and 30 kg/m^2^; (2) not currently pregnant; (3) not taking any medications or dietary supplements know to affect body composition, as indicated by the product documentation; and (4) premenopausal status, confirmed by self-report–defined as experiencing menstruation lasting ≤7 days within a cycle length of 22–35 days.^40^

Participants were excluded if they: (1) had been diagnosed by a physician with cardiovascular or neurological diseases; (2) presented signs or symptoms potentially indicative of cardiovascular or pulmonary disorders, including but not limited to: chest pain (with or without radiation to the jaw, neck, or arms) related to exertion; shortness of breath at rest or during mild exertion; dizziness, history of fainting, or syncope during physical activity; orthopnea; bilateral leg edema; awareness of a rapid or forceful heartbeat at rest or during minimal exertion; or intermittent claudication during activities such as walking or cycling; or (3) has any musculoskeletal or orthopedic problems–such as joint deformities or persistent joint pain that could interfere with their ability to participate in the study.

All participants provided written informed consent prior to data collection. The study protocol was approved by the Ethical Review Sub-Committee Board for Human Research Involving Sciences, Thammasat University, No.3 following the Declaration of Helsinki (COA No. 075/2565).

### 2.2 Measures

#### 2.2.1 Brachial-ankle pulse wave velocity (baPWV) and ankle-brachial index (ABI)

Brachial-ankle pulse wave velocity (baPWV), an index of arterial stiffness, and ankle-brachial index (ABI), an index of peripheral arterial stenosis were assessed using the VP-1000 machine (Omron Healthcare, Kyoto, Japan). The Omron device is equipped with four blood pressure cuffs placing at wrists and ankles, electrocardiogram, and phonocardiogram. The device measures calculates baPWV, a widely used indicator of arterial stiffness especially in East Asian countries.^41^ The travel distance for baPWV calculation is automatically estimated based on the participant's height. The device also measures systolic and diastolic blood pressure at the brachial artery and posterior tibial artery to compute the ABI. Participants were asked to rest in a supine position for 15 min in a quiet, temperature-controlled room before the measurement began. Each measurement was taken three times, and the average value was used for analysis. Values obtained from the left side of the body were used for all analyses.

#### 2.2.2 Carotid intima-media thickness (cIMT)

cIMT was assessed using ultrasound imaging acquired with a Philps EPIQ 5 Ultrasound System (Philips Healthcare), which was equipped with a high-resolution linear-array transducer (12–5 MHz). Longitudinal images were captured from the straight segment of the common carotid artery, proximately 1–2 cm proximal to the bifurcation. The ultrasound images were recorded and analyzed using specialized automated software (Carotid Analyzer; Medical Imaging Applications, Coralville, IA). IMT was defined as the distance between the leading edge of the lumen–intima interface and the media–adventitia interface on the far wall of the artery.^42^

#### 2.2.3 Maximal oxygen consumption

A graded exercise test was performed on a motor-driven treadmill. After a 3-min warm-up at a speed of 3.3 mph and slope 0 %grade, participants began walking or running at the same speed. Following the warm-up period, the treadmill incline was progressively increased by 1 % every minute. Maximal effort during the test was confirmed when at least two of the following criteria were met: (1) a plateau in VO_2_, (2) respiratory exchange ratio (RER) ≥ 1.15, (3) rating of perceive exertion of >17 on a scale 6–20, or (4) achievement of at least 95 % of the age-predicted maximal heart rate, calculated using Tanaka's equation (208 – 0.7 x age).^43^ Gas exchange was assessed throughout the test using breath-by-breath cardiopulmonary gas exchange system (MetaMax 3B, Cortex).

#### 2.2.4 Body composition

Body composition was assessed using dual-energy X-ray absorptiometry (DXA) (Lunar Prodigy, G.E. Medical System, Fairfield, CT). Whole-body scans were analyzed to quantify fat, and lean tissue across specific body regions. Data were processed using enCORE software version 13.60. To evaluate android fat, a region of interest was automatically defined, with the lower boundary (caudal limit) positioned at the top of the iliac crest and the base of the skull, determining its upper boundary (cephalad limit). Within this android region, both abdominal and subcutaneous fat and visceral fat were estimated. Visceral fat mass was calculated by subtracting subcutaneous fat from the total fat in the android region. DXA has been widely validated as a sensitive and cost-effective method for assessing visceral fat, with results comparable to those obtained via computed tomography^44^^,^^45^ which is considered the gold standard for measuring visceral fat.

### 2.3 Procedures

All testing was performed in the morning after the participants had fasted overnight for at least 12 h. The participants were asked not to perform exercise at least 24 h before the testing day.

To minimize potential confounding effects from acute exercise, the exercise test was always conducted as the final assessment of the session. All other measurements were allowed to perform in varying order depending on participant flow and equipment availability, as these were not expected to significantly influence one another.

### 2.4 Statistical analysis

All statistical analyses were conducted using IBM SPSS version 29 (IBM, Armonk, NY, USA). The normal distributions of all variables were first examined using the Shapiro-Wilk test of normality. Partial correlation analyses controlling for age were performed to assess the relationship among body composition indices (i.e., BMI, percentage of body fat, visceral adipose tissue mass, and lean mass), maximal oxygen consumption, and arterial health variables, as age is a known confounding factor that independently influences both vascular function, cardiorespiratory fitness, and body composition.^46^, ^47^, ^48^, ^49^, ^50^, ^51^ A two-tailed α-level of .05 (p < 0.05) was set *a priori* as the criterion for statistical significance.

Significant relationships identified through partial correlation, whether between cardiorespiratory fitness and body composition indices or between body composition and vascular health indices, were further analyzed to assess the mediating effect of body composition on the relationship between cardiorespiratory fitness and vascular health. Structural equation modeling (SEM) was used to test the mediating role of body composition on the association between maximum oxygen consumption and vascular variables. SEMs were analyzed using AMOS version 29 (IBM, Armonk, NY, USA). Bias-corrected bootstrap analyses were conducted with 5000 bootstrap samples to assess the significance of indirect (mediated) effects. An indirect effect was considered statistically significant if the 95 % confidence interval (CI) did not include zero.^52^ The proportion of mediation was calculated by dividing the indirect effect by the total effect, representing the relative contribution of the mediating pathway to the overall relationship.

The sample size was calculated using G∗Power software version 3.1.9.7, based on the correlation coefficient reported in a study by Tomoto T et al.,^53^ which investigated similar variables. Specifically, the correlation between baPWV and maximal oxygen consumption from Tomoto's study was use as the reference value for calculation. The statistical power was set at 80 %, and the calculation indicated that a minimum sample size of 50 participants was required.

## 3 Results

### 3.1 Participant characteristics

The average age of participants (n = 67) was 30 ± 9 years with normal blood pressure. Average BMI was defined as overweight for Asian population.^54^ Other participant characteristics are presented in Table 1.

**Table 1:** Characteristics of the participants (n = 67).

	Mean	SD
Age (years)	30.9	9.5
Weight (kg)	63.8	14.1
BMI (kg/m^2^)	24.7	5.1
Brachial systolic blood pressure (mmHg)	107.8	9.8
Brachial diastolic blood pressure (mmHg)	65.0	7.4
Brachial pulse pressure (mmHg)	42.8	5.0
baPWV (cm/sec)	1110.74	149.26
ABI	1.13	.1
cIMT (mm)	.43	.04
Percentage of body fat	39.2	6.1
Visceral adipose tissue mass (g)	518.6	409.7
Lean mass (g)	35355.51	8617.37
Maximal oxygen consumption (ml/kg/min)	29.66	5.6
Percent Maximal heart rate achieved during graded exercise testing (% of age-estimated maximum heart rate)	95.7	5.9
RER at peak exertion during graded exercise testing	1.26	.1

BMI = body mass index; baPWV = brachial-ankle pulse wave velocity; ABI = ankle-brachial index; cIMT = carotid intima media thickness; RER = respiratory exchange ratio.

### 3.2 Associations of body composition, arterial health, and cardiorespiratory fitness

Partial correlation coefficients of all the variables measured can be found in Table 2. Partial correlation analyses reveal significant relationship between BMI and brSBP (r = .484, p < 0.001), brPP (r = .366, p = 0.003), and cIMT (r = .273, p = 0.029). Percentage of body fat showed significant associations with brSBP (r = .465, p < 0.001), brPP (r = .356, p = 0.004), brPWV (r = .257, p = 0.041), and cIMT (r = .253, p = 0.043) after controlling for age. The partial correlation between visceral adipose tissue mass and vascular function indices demonstrated significant relationships between this body composition parameter and brSBP (r = .582, p < 0.001), brPP (r = .401, p = 0.001), and baPWV (r = .256, p = 0.034).

**Table 2:** Partial correlation coefficients (r) between body composition-related variables, cardiorespiratory fitness, and vascular health-related variables, controlling for age.

	BMI	Percentage of body fat	Visceral adipose tissue mass	Lean body mass	Maximal oxygen consumption	brSBP	brPP	baPWV	ABI	cIMT
**BMI**	1									
**Percentage of body fat**	**.783∗∗**	1								
**Visceral adipose tissue mass**	**.866∗∗**	**.744∗∗**	1							
**Lean body mass**	**.504∗∗**	.216	**.261∗**	1						
**Maximal oxygen consumption**	**−.481∗∗**	**−.529∗∗**	**−.508∗∗**	−.055	1					
**brSBP**	**.484∗∗**	**.465∗∗**	**.582∗∗**	.144	**−.317∗**	1				
**brPP**	**.366∗**	**.356∗**	**.401∗**	.244	−.204	**.730∗∗**	1			
**baPWV**	.131	**.257∗**	**.265∗**	−.060	−.125	**.702∗∗**	**.455∗∗**	1		
**ABI**	.226	.106	.185	.135	.062	.036	−.015	−.025	1	
**cIMT**	**.273∗**	**.253∗**	.144	.186	−.237	**.283∗**	**.447∗∗**	.080	−.049	1

∗*P* < 0.05.

∗∗*P* < 0.001.

BMI = body mass index; brSBP = brachial systolic blood pressure; brPP = brachial pulse pressure; baPWV = brachial-ankle pulse wave velocity; ABI = ankle-brachial index; cIMT = carotid intima media thickness.

Cardiorespiratory fitness as measured by maximal oxygen consumption revealed significant relationship with BMI (r = −.481, p < 0.001), percentage of body fat (r = −.529, p < 0.001), VAT mass (r = −.508, p < 0.001), and brSBP (r = −.317, p = 0.011) after controlling for age.

However, after controlling for age, partial correlation analysis revealed no significant associations between ABI and any of the body composition parameters, nor between ABI and maximal oxygen consumption.

### 3.3 Mediation analysis of body composition indices on the relationship between cardiorespiratory fitness and vascular health

Based on the significant associations observed in the partial correlation analysis, SEM was conducted to further examine the mediating effects of body composition on the relationship between cardiorespiratory fitness and arterial health.

**Fig. 1:**
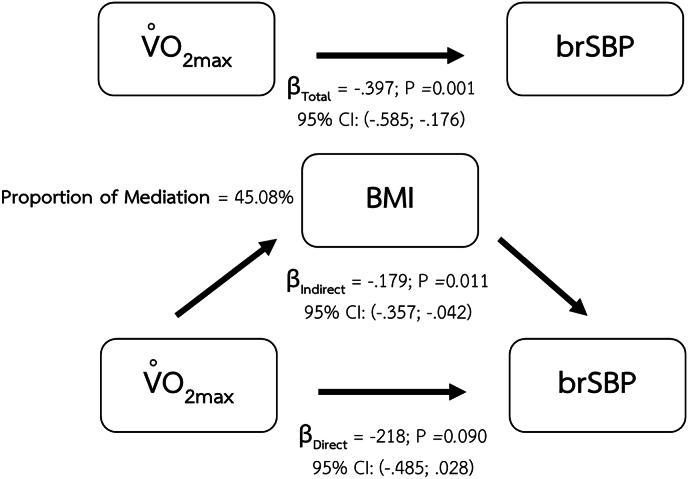
Mediating effect of BMI on the relationship between CRF and brSBP.

**Fig. 2:**
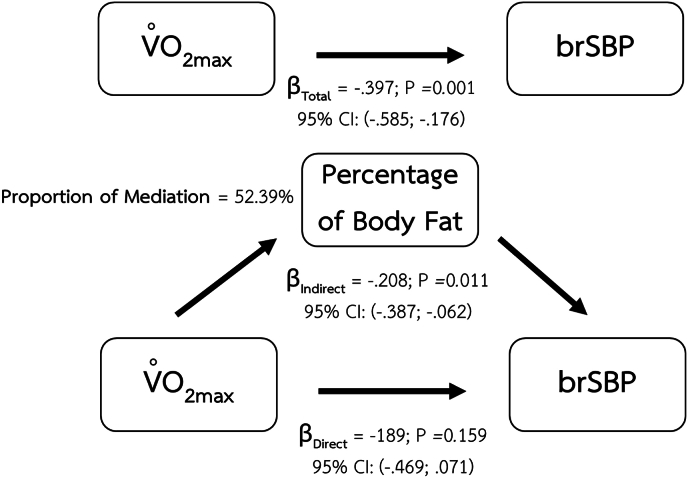
Mediating effect of percentage of body fat on the relationship between CRF and brSBP.

**Fig. 3:**
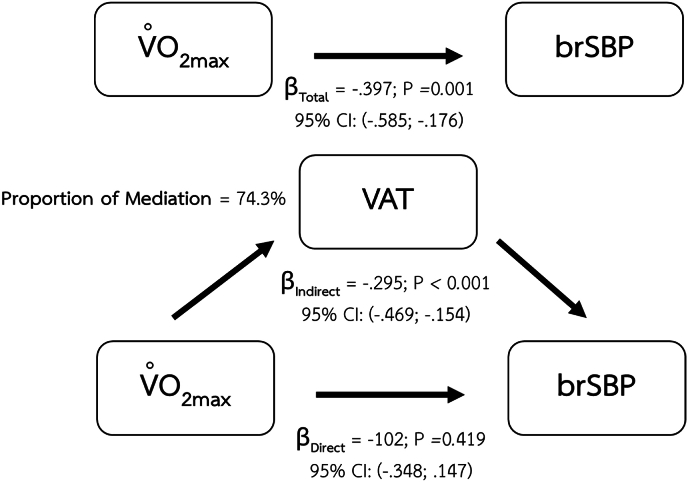
Mediating effect of VAT on the relationship between CRF and brSBP.

The mediation analysis revealed that the relationship between maximum oxygen consumption and brSBP was mediated by BMI (Fig. 1), percentage of body fat (Fig. 2), and VAT mass (Fig. 3).

**Fig. 4:**
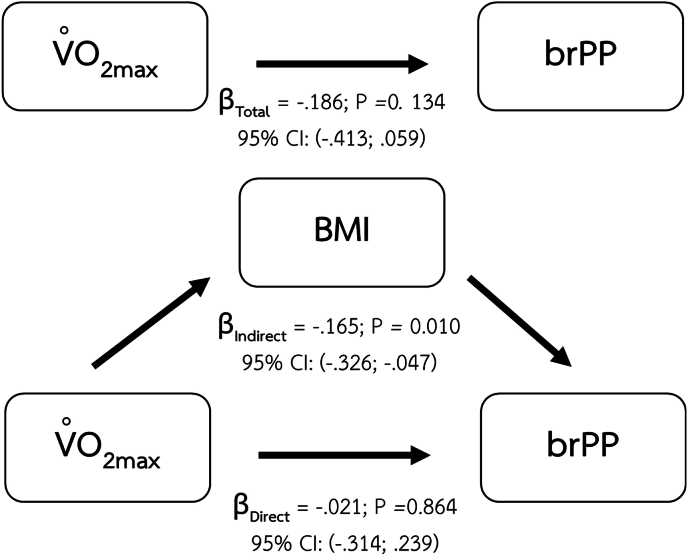
Mediating effect of BMI on the relationship between CRF and brPP.

**Fig. 5:**
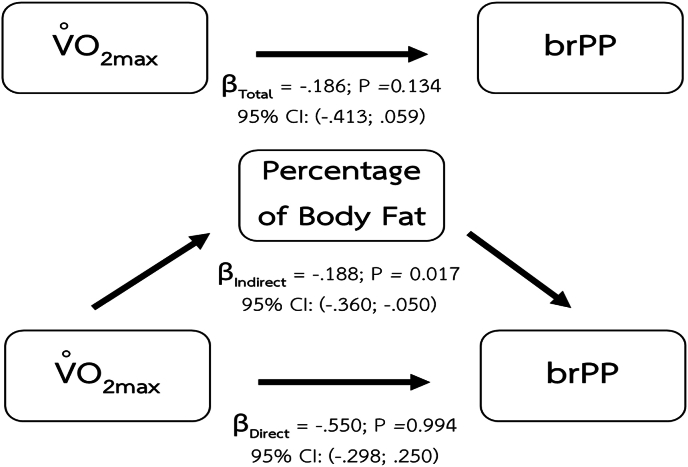
Mediating effect of Percentage of Body Fat on the relationship between CRF and brPP.

**Fig. 6:**
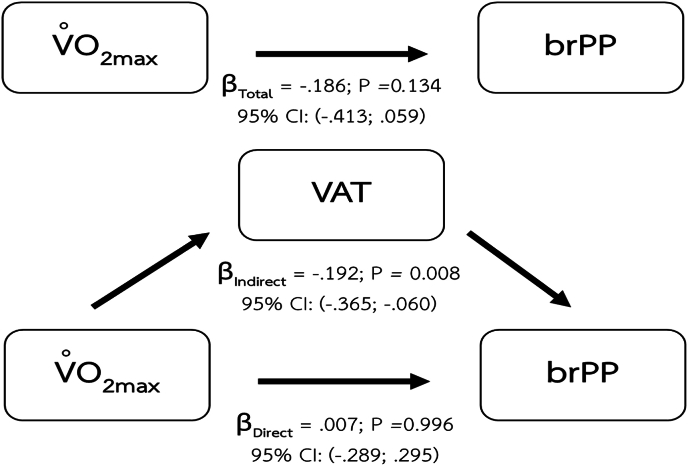
Mediating effect of VAT on the relationship between CRF and brPP.

We found that the indirect effects of body composition also play a role in the relationship between maximum oxygen consumption and brPP. The body composition parameters that demonstrate the mediating effect include BMI (Fig. 4), percentage of body fat (Fig. 5), and VAT mass (Fig. 6).

**Fig. 7:**
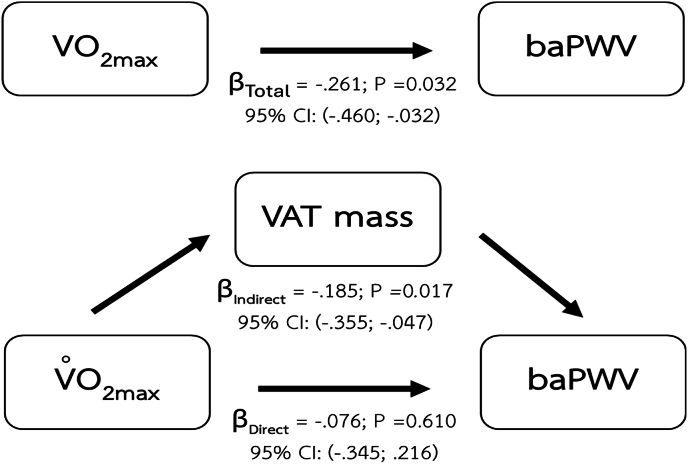
Mediating effect of visceral adipose tissue mass (VAT mass) on the relationship between cardiorespiratory fitness and baPWV.

Furthermore, the result shows that VAT mass mediated the relationship between maximal oxygen consumption and baPWV (Fig. 7).

## 4 Discussion

Major findings of the present study are the mediating effects of body composition parameters including BMI, percentage of body fat, and VAT mass on the relationship between CRF and arterial health indices. The significant mediating role of BMI shows in the relationship between CRF and vascular health parameters including brSBP, brPP. Percentage of body fat also demonstrates its mediating effect on the same relationships as BMI. However, visceral adipose tissue was the only body composition index that demonstrated a mediating effect on the relationship between CRF and baPWV.

### 4.1 Mediating effect of BMI and percentage of body fat on the relationship between CRF and vascular health indices

The potential mediating role of BMI and percentage of body fat in the relationship between CRF and brSBP is supported by a large prospective study in women conducted by Shuger et al., in 2008.^8^ In this longitudinal study, with an average follow-up duration of 16.7 ± 7.8 years, higher BMI and percentage of body fat were significantly associated with the incidence of hypertension reported at follow-up. A recent study in Chinese population also reports a significant positive association between brachial blood pressure and BMI.^55^ These findings might infer that any change in BMI and percentage of body fat could lead to an increase in blood pressure. Improved CRF may contribute to a reduction in BMI and percentage of body fat, which in turn can lead to favorable changes in blood pressure. Regular aerobic exercise enhances energy expenditure and promotes fat oxidation, thereby could contribute to reducing fat accumulation and eventually improving body composition especially BMI, and percentage of body fat.^56^

The absence of a mediating effect of BMI on the relationship between CRF and baPWV in our study may be supported by a recent study in a Chinese population, which reported a linear relationship between BMI and baPWV only among women age over 50 years.^55^

### 4.2 Mediating effect of VAT mass on the relationship between CRF and vascular health indices

The current study demonstrates the mediating effect of VAT mass on the relationship between CRF and arterial health indices including brSBP, brPP, and baPWV. The role of VAT as a mediator on the association between CRF and arterial health indices could be supported by the recent study that show the influence of VAT on the effect of insulin resistance on arterial stiffness.^57^ As the progression of arterial stiffness involves a multifaceted interplay among hormonal influences, adipose tissue-derived cytokines, and the dynamic interactions between vascular cell, the extracellular matrix, perivascular adipose tissue, and immune cells within the vascular environment these complexity could be the reason that we can detect the mediating effect of visceral tissue on baPWV, a surrogate of arterial stiffness without any significant mediating effects of other two body composition metrics (i.e., BMI, and percentage of body fat). As this arterial stiffness index captures more arterial tree (i.e., elastic artery and muscular artery), it only shows the association with the visceral adipose tissue that could exert more extent effect compared to BMI and percentage of body fat. Another reason might involve the cascade of lipid accumulation in the body. At the earliest, excess lipids are deposited in subcutaneous region which can technically detect the change of body composition by the change in BMI, and the percentage of body fat. With a continuous lipid accumulation that exceeds the capacity of the subcutaneous sink, the extra lipid moves to store between the visceral organs. Visceral adipose tissues have a greater ability to secrete substances that cause the damage of vasculature.

Excess adiposity was closely related to vascular dysfunction. One of the mechanisms involved in adiposity-related vascular dysfunction were adipokines, the compounds those be secreted by adipose tissues. Adipokines which could affect arterial structure and function via multiple mechanisms such as induction of inflammation, stimulation of vascular smooth muscle cell proliferation, and activation of sympathetic activity.^58^^,^^59^ Previous studies have suggested relations between adipokines and arterial stiffness^60^, ^61^, ^62^ as we also demonstrated in the present study that there is a significant relationship between baPWV, an index of arterial stiffness, and VAT mass even after age was taken into account.

### 4.3 Lipid-driven metabolic alterations and segmental vascular dysfunction

Metabolic alterations driven by lipid dysregulation contribute to vascular dysfunction through a progressive and multi-stage pathophysiological cascade. This process often begins with elevated circulating lipids–particularly oxidized low-density lipoprotein (oxLDL)–which interact with endothelial cells via receptors such as LOX-1. These interactions initiate a cascade of oxidative stress, increased production of pro-inflammatory cytokines, and reduced nitric oxide bioavailability, ultimately leading to endothelial dysfunction and increased arterial tone.^63^^,^^64^

As metabolic overload persists, excess lipids are initially stored as subcutaneous adipose tissue, which serves as a protective metabolic sink. However, once adipose tissue expands excessively and begins to exceed its functional capacity, the lipids are redirected to ectopic depots, particularly visceral adipose tissue. Visceral adipose tissue is more metabolically active and pro-inflammatory than subcutaneous adipose tissue. Visceral adipose tissue secretes bioactive molecules such as tumor necrosis factor-α (TNF-α), interleukin-6 (IL-6), and angiotensinogen, which collectively promote vascular inflammation, endothelial dysfunction, and increased arterial stiffness.^65^, ^66^, ^67^

The differential mediating effects observed in our study may be explained by distinct stages of lipid accumulation and their respective vascular consequences. Our findings demonstrated that BMI and percentage of body fat mediated the relationship between CRF and brSBP as well as brPP–both of which reflect hemodynamic changes in more muscular arteries. These body composition indices, which largely capture overall and subcutaneous fat accumulation, may reflect earlier metabolic alterations that primarily influence vascular tone and endothelial function. In contrast, only VAT mass mediated the relationship between CRF and baPWV, a measure of systemic arterial stiffness involving both elastic and muscular arteries.^4^ This suggests that more advanced stages of lipid accumulation, particularly ectopic fat deposition in visceral depots, may exert broader and more deleterious effects on vascular integrity and stiffness, thereby affecting a wider segment of the arterial tree. Such findings align with the notion that subcutaneous fat serves as a temporary metabolic buffer, while visceral adiposity is more strongly linked to vascular remodeling and long-term structural changes.^68^^,^^69^

Regarding parameters indicative of arterial stenosis, including cIMT and ABI, no significant mediating effects of body composition indices were observed in the relationship between these arterial narrowing markers and cardiorespiratory fitness. This may be attributed to the fact that structural changes in the arterial wall, such as stenosis, typically develop over a longer period and may not be as immediately responsive to metabolic alterations as functional vascular changes, such as those involving vascular smooth muscle tone.

### 4.4 Not only adipokines but also myokines play a crucial role in mediating the vascular benefits of aerobic exercise

Although aerobic exercise has well-established evidence supporting its beneficial effects on vascular function, the observed vascular improvements are not fully explained by changes in body composition, particularly lipid-related variables. The presence of only partial mediating effect suggests that other biological mechanisms may be involved in the protective effects of aerobic exercise. Beyond the influence of adipokines, emerging research highlights the role of myokines, such as follistatin-like 1 (FSTL-1), which is released from skeletal muscle during exercise. FSTL-1 has been shown to exert protective effects on endothelial function, contributing to vascular health independently of lipid metabolism.^70^^,^^71^

### 4.5 Limitation of the study

The nature of study design could not provide the causal relationship between arterial stiffness and visceral adipose tissue. Further longitudinal research could provide more insights regarding the potential of adiposity-mediating effect on the relationship between aerobic capacity and vascular function. In addition to the observational design, which precludes definitive causal inference, several other limitations should be acknowledged. First, the relatively small sample size may limit the statistical power to detect weaker associations or interactions. Second, our participants were relatively homogeneous in terms of health status and environmental factors, which may limit the generalizability of our findings to broader populations. Third, although we accounted for age which is the important covariate for the parameters of interest for the study, unmeasured confounding variables such as dietary intake, and sleep patterns may have influenced the observed associations.

### 4.6 Implications for Women's health before menopause

The premenopausal period represents a critical window for cardiovascular disease prevention, as hormonal changes associated with menopause are known to adversely affect lipid metabolism and vascular function. Our findings emphasize the importance of maintaining optimal cardiorespiratory fitness and a healthy body composition prior to menopause to preserve arterial health. Aerobic exercise, which improves cardiorespiratory fitness and promotes favorable adaptations in fat metabolism, may enhance lipid oxidation and reduce the secretion of pro-inflammatory adipokine, thereby contributing to improve arterial health. This link underscores the importance of promoting aerobic fitness in younger women not only for weight management but also for vascular protection, through enhanced lipid handling and consequent reduction in adiposity.

### 4.7 Future directions

While this study contributes novel insights into the mediating role of body composition in arterial health, several areas warrant further investigation. First, longitudinal studies are needed to establish the temporal and causal relationship among cardiorespiratory fitness, body composition, and arterial stiffness in women across different reproductive stages. Second, interventional studies assessing whether improvements in cardiorespiratory fitness through structured aerobic training lead to parallel changes in body composition, especially those parameters related to lipid metabolism. Lastly, integrating biomarkers of inflammation and plasma lipid profiles could further elucidate the mechanistic pathways linking cardiorespiratory fitness, adiposity, and arterial function.

## 5 Conclusion

The present study highlights the importance of the interplay among factors already known to influence arterial health. Our findings suggest that body composition metrics such as BMI, percentage of body fat, and VAT mass may serve as key outcomes to monitor when exercise intervention are designed to improve arterial health in premenopausal women.

## Data availability statement

The raw data supporting the conclusions of this article will be made available by the authors, without undue reservation.

## Ethics statement

The studies involving human participants were reviewed and approved by the Ethical Review Sub-Committee Board for Human Research Involving Sciences, Thammasat University No.3 (COA No. 075/2565).

## Author contributions

JL, NC, PO, TS, SP, and PN contributed substantially to the literature search and study design. JL, NC, PO, TS, SP, and PN provided data collection. JL, and NC contributed to the data analysis, interpretation, and manuscript preparation. All authors critically reviewed the manuscript.

## Funding

The financial support provided by the Faculty of Allied Health Sciences Research Fund, Thammasat University (Contract No. AHSRS4/2565).

## Declaration of interest

The authors declare that there are no competing financial interests or personal relationships that could have appeared to influence the results reported in the manuscript.
